# Silver Nanoparticle-Based Finishing for Leather Antimicrobial and UV Protection

**DOI:** 10.3390/mi16040376

**Published:** 2025-03-26

**Authors:** Claudia Cirillo, Mariagrazia Iuliano, Francesca Fierro, Claudia Florio, Gaetano Maffei, Andrea Loi, Todor Batakliev, Maria Sarno

**Affiliations:** 1 Department of Physics “E.R. Caianiello”, University of Salerno, Via Giovanni Paolo II, 132, 84084 Fisciano, Italy; maiuliano@unisa.it (M.I.);; 2 Centre NANO_MATES, University of Salerno, Via Giovanni Paolo II, 132, 84084 Fisciano, Italy; 3 Stazione Sperimentale per l’Industria delle Pelli e delle Materie Concianti—SSIP (Italian Leather Research Institute), Comprensorio Olivetti, Via Campi Flegrei, 34, 80078 Pozzuoli, Italy; 4 Conceria DMD SOLOFRA S.p.A., Via Celentane, 9, 83029 Solofra, Italy; 5 Mario Levi Italia S.r.l., Via Arzignano, 130, 36072 Chiampo, Italy; 6 Open Laboratory on Experimental Micro and Nano Mechanics (OLEM), Institute of Mechanics, Bulgarian Academy of Sciences, Acad. G. Bonchev Str., Block 4, 1113 Sofia, Bulgaria

**Keywords:** leather-finishing agents, Ag-F NPs, ligand exchange process, UV protection, antimicrobial activity

## Abstract

This study focuses on preparing and characterizing functionalized silver nanoparticle-based (Ag-F NPs) finishing agents for leather treatment. Ag-F NPs were synthesized and functionalized through a ligand exchange process with citric acid, enhancing their dispersion stability in aqueous media. The nanoparticles were incorporated into polyurethane- and nitroemulsion-based finishing formulations and applied to ovine and bovine leather via a spray coating process. Morphological (SEM, TEM), structural (XRD), thermal (TGA), and spectroscopic (FT-IR) analyses confirmed successful functionalization and uniform dispersion within the finishing layer. Leather samples treated with Ag-F NPs exhibited a significant improvement in antibacterial properties, with microbial growth reduction of up to 90% after 72 h. Additionally, accelerated aging tests demonstrated enhanced UV resistance, with a 30% lower color change (∆E) compared to control samples. The Ag-F NPs-based finishing layers also exhibited superior abrasion and micro-scratch resistance, maintaining a stable coefficient of friction over time. These findings demonstrate the potential of Ag-F NPs as multifunctional leather-finishing agents, making them highly suitable for applications in the automotive, footwear, and leather goods industries.

## 1. Introduction

Leather finishing is a crucial phase in leather processing, essential for refining both the aesthetic and functional attributes of the material. This phase aims to enhance the visual appeal, tactile feel, and overall performance of leather, including critical properties such as mechanical durability [1], water repellency [2], microbial resistance [3], and ultraviolet (UV) protection [4]. This operation can be carried out using mechanical or chemical methods, with chemical treatments being widely adopted for surface protection, color enhancement, and defect masking. Traditional leather finishing typically involves polymeric coatings, such as polyurethane [5] and nitrocellulose emulsions [6], widely favored for flexibility, surface uniformity, and enhanced mechanical properties [1]. Nevertheless, while these conventional finishes provide satisfactory baseline performance, they often fall short in delivering multifunctional properties. In particular, there is a growing demand for coatings that not only preserve leather aesthetics and durability but also provide advanced antimicrobial protection [7], long-lasting UV resistance, and sustained mechanical performance under prolonged usage conditions [8].

In recent years, nanotechnology has gained increasing interest due to its potential to improve both mechanical performance and versatility [9,10,11]. The incorporation of nanoparticles into finishing formulations has shown considerable promise in enhancing surface adhesion, reinforcing mechanical properties, and introducing novel functionalities, such as antibacterial activity and self-cleaning behavior [12,13]. Despite extensive research on functional enhancements [12,13,14], large-scale industrial adoption remains challenging due to the limited availability of systematic studies addressing their practical implementation. Key challenges include maintaining nanoparticle dispersion stability in finishing formulations, ensuring compatibility with existing production processes, and preserving the leather’s visual and mechanical properties. Overcoming these barriers requires closer collaboration with manufacturers and the development of scalable, cost-effective solutions.

Among the various nanomaterials, silver nanoparticles (Ag NPs) stand out due to their exceptional physicochemical properties, including strong antimicrobial activity [15,16], UV protection capabilities [16], and reinforcement of mechanical integrity [17], unique optical effects, and catalytic potential. In recent years, Ag NPs have been extensively studied in the textile and leather industries for their ability to provide multifunctional benefits, particularly in enhancing antimicrobial resistance, UV protection, and self-cleaning capabilities [18,19,20,21,22]. Their localized surface plasmon resonance (LSPR) makes them particularly advantageous, as it allows for both coloration enhancement and antibacterial properties, offering a dual benefit in high-quality leather production.

The potential applications of Ag NPs in leather finishing have been primarily explored for their antimicrobial effects, given that leather products, such as footwear, garments, and accessories are often exposed to environments that promote bacterial proliferation. The fibrous collagen structure of leather readily absorbs moisture, oxygen, and organic residues from skin contact, creating an ideal surface for bacterial adhesion and growth [23,24,25]. This can result in issues such as unpleasant odors, discoloration, degradation of mechanical properties, and even skin irritation or infections. To mitigate these concerns, research has focused on the development of antibacterial coatings for leather, with Ag NPs emerging as a particularly effective solution. Their mode of action involves the release of silver ions (Ag^+^), which disrupt microbial membranes, interfere with cellular processes, and prevent bacterial proliferation [26,27,28,29,30,31].

In this study, we introduce the integration of silver nanoparticles into finishing coatings to impart advanced multifunctional properties to leather. By optimizing precise functionalization techniques, we improved nanoparticle dispersion stability within finishing formulations while simultaneously reinforcing the mechanical durability of the coating. The nanoparticles were specifically designed and tested to enhance aging resistance, abrasion resistance, and antimicrobial performance, ensuring long-term functionality.

To achieve a uniform and consistent deposition of the finishing film in a single-step application capable of delivering multiple functionalities simultaneously, we placed particular emphasis on formulation stability and compatibility with standard finishing processes. A cost-effective and scalable synthesis approach was selected for nanoparticle production, enabling practical large-scale application during the finishing stage of goat, bovine, and sheep leather. This strategy is particularly well-suited for industries requiring high-performance materials, such as automotive, footwear, and luxury leather goods.

## 2. Experimental

### 2.1. Materials

The leather used consisted of pink crust sheepskin received from DMD Tannery Solofra S.p.A. (Solofra, Italy). Benzyl ether, silver nitrate (AgNO_3_), 1,2-hexadecanediol, oleic acid (OA), citric acid (CA), methanol, ethanol, and hexane, as well as other chemicals, were purchased from Sigma-Aldrich (St. Louis, MO, USA). For the application of nanoparticles in the leather-finishing process, polyurethane-based finishing agents (code 5766, RODA FIX 5766 GLOSS, TFL Italia S.p.A., Buscate, Italy) and nitroemulsion (code 707, FYL WAX 707, GSC Group S.p.A., Montebello Vicentino, Italy) were used.

### 2.2. Synthesis of Functionalized Silver Nanoparticles (Ag-F NPs)

The fabrication of Ag-F NPs consists of two main stages: synthesis, as reported by Sarno et al. [32], and subsequent functionalization with citric acid [33]. Briefly, for the synthesis process, the following chemicals were used: benzyl ether (0.020 L), AgNO_3_ (0.002 mol), 1,2-hexadecanediol (0.010 mol), and OA (0.012 mol). These reagents were combined in a batch reactor and subjected to continuous magnetic stirring under a nitrogen atmosphere. The temperature gradually increased from 25 °C to 200 °C over two hours, followed by a further increase to 285 °C for an additional hour. Once the synthesis was complete, the resulting mixture underwent a series of purification steps via centrifugation (7500 rpm, 30 min) using ethanol and hexane. This washing process was repeated three to four times to remove any residual impurities. The final product was then left to dry at room temperature for 24 h. Subsequently, to functionalize the Ag NPs, 0.1 g of Ag NPs was mixed with 1.0 g of CA in 40 mL of methanol. This mixture was then subjected to sonication for 10 min at maximum ultrasonic power (UP400S—Hielscher, Teltow, Germany). As the process progressed, the solution’s color gradually changed from black to a dark transparent shade. The NPs were then washed multiple times with ethanol and hexane, followed by centrifugation at 7500 rpm for 5 min. This purification step, repeated two to three times, ensured the removal of any excess ligands. The resulting hydrophilic NPs exhibited excellent dispersibility in water and remained stable in other hydrophilic media over time.

### 2.3. Ag-F NPs Leather-Finishing Agents’ Preparation

Ag-F NPs leather-finishing agents were prepared using the procedure reported by Iuliano et al. [34]. Briefly, a certain quantity of Ag-F NPs was dispersed in 15 mL of deionized water through ultrasonic treatment for 15 min, using an ultrasound tip at maximum power of ultrasound (UP400S—Hielscher). This ultrasonic treatment effectively minimized nanoparticle agglomeration, ensuring stable and homogeneous aqueous suspensions, as previously reported [34,35], due to its ability to generate strong mechanical forces that separate agglomerates and ensure uniform particle distribution in aqueous media. Subsequently, the nanoparticle dispersion was thoroughly mixed with 15 mL of PU5766 or 707 to obtain Ag-F-PU5766 or Ag-F-707 composite leather-finishing agents. The mixing was performed under gentle stirring for approximately 10 min at room temperature to ensure complete integration and stability of nanoparticles within the finishing formulations.

After mixing, visual inspections confirmed the high stability of the nanoparticle dispersions within both finishing formulations, observing no sedimentation or visible aggregation even after prolonged storage.

### 2.4. Leather Finishing

The pink crust sheepskins were finished with an Ag-F-PU5766 or Ag-F-707 finishing agent, which had been previously prepared, via spray application onto the leather surface [12]. The leather samples measured 20 cm × 30 cm. The control sample was treated with the finishing composite without nanoparticles.

### 2.5. Characterization of Nanoparticles and Leather-Finishing Agents

The nanoparticles were structurally and morphologically characterized using various analytical techniques. X-ray diffraction (XRD) measurements were carried out using a Bruker D2 diffractometer (Billerica, MA, USA) with CuKα radiation. Scanning electron microscope (SEM) images were acquired with a TESCAN-VEGA LMH (Brno, Czechia) operating at 230 V, equipped with an energy dispersive X-ray spectroscopy (EDX) probe. Transmission electron microscope (TEM) images were obtained using an FEI Tecnai (Hillsboro, OR, USA) microscope operating at 200 keV, equipped with a LaB_6_ filament as the electron source and an EDX probe. Nanoparticle tracking analysis (NTA) was conducted using a Malvern NanoSight LM10 from Malvern Instruments (Malvern, UK). Furthermore, the characterization of nanoparticles and leather-finishing agents was performed using thermogravimetric analysis (TG-DTG) and Fourier transform infrared spectroscopy (FT-IR). Thermogravimetric analysis was carried out using a TGA 2 METTLER TOLEDO (Columbus, OH, USA) instrument under airflow at a heating rate of 10 °C/min. FT-IR spectra were recorded using a Nicolet^TM^ iS50 FT-IR spectrometer (ThermoFischer Scientific, Waltham, MA, USA).

### 2.6. Characterization of Leather Properties

#### 2.6.1. Antimicrobial Properties Analysis

The antimicrobial properties were evaluated using the TTC/MALT dipslide test [12]. In this procedure, leather samples (20 cm^2^ in size), sourced from automotive and leather goods applications, were impregnated on the grain side with the culture medium matrix mentioned above. The dipslides containing the contaminated culture medium were then incubated in an oven at 36 °C to assess bacterial growth. The bacterial population, expressed as colony forming units per milliliter (CFU/mL), was measured at 24, 48, and 72 h.

#### 2.6.2. Aging Tests

Accelerated sunlight resistance tests were conducted on leather samples following a standard finishing process by DMD SOLOFRA S.p.A., Solofra (AV), Italy. Specifically, a paint spray gun operating at 5 atm was used to apply 10 mL of the finishing mixture onto each leather sample, ensuring complete surface coverage. After drying, the samples were cut, mounted on a black card, and exposed to a Xenon lamp (Solarbox 1500 Standard) at 50 °C for 24 and 48 h. A section of the sprayed samples was covered to prevent exposure to the Xenon lamp, serving as a reference for comparison. The evaluation of discoloration in sheep and goat leathers used for footwear and leather goods was performed following the ISO 105 B02 standard [36].

Additionally, accelerated aging tests were conducted on sheep and bovine leather samples designated for various applications (footwear and leather goods, respectively). Further aging tests were performed on leather samples intended for the automotive sector by Mario Levi Italia S.r.l., Chiampo (VI), Italy—using a CGOLDENWALL YS3010 Spectrophotometer Color Matching Colorimeter. These tests followed the SAE J-2412 method [37], which involves exposing the samples to UV radiation and visible light generated by a xenon arc within a controlled radiation chamber. The procedure also incorporates a dark cycle under high-humidity conditions. The tests were conducted at irradiance levels of 225 KJ/m^2^ and 601 KJ/m^2^.

#### 2.6.3. Abrasion Resistance Tests and Wear/Micro-Scratch Measurements

Abrasion resistance tests were carried out on automotive leather samples following ISO 17076 standards—part 1 (Taber method) [38] and part 2 (Martindale ball plate) [39]. Since the finishing application was performed at a laboratory scale without a pre-primer, the samples underwent 100 cycles, a lower number than specified in official standards and regulations. Micro-scratch and wear tests were conducted using a UMT-2 tribometric system (Bruker, Billerica, MA, USA). The micro-scratch test was performed with a diamond conical stylus featuring a 5 μm tip radius, sliding under a progressively increasing linear load ranging from 50 mN to 300 mN. The wear test, on the other hand, involved the reciprocating motion of a chromium stainless steel ball (6.35 mm in diameter) over the specimen’s surface with a normal force of 2 N applied for 300 s.

## 3. Results and Discussion

### 3.1. Nanoparticles Characterization

#### 3.1.1. Chemical–Physical Characterization of Nanoparticles

**Figure 1:**
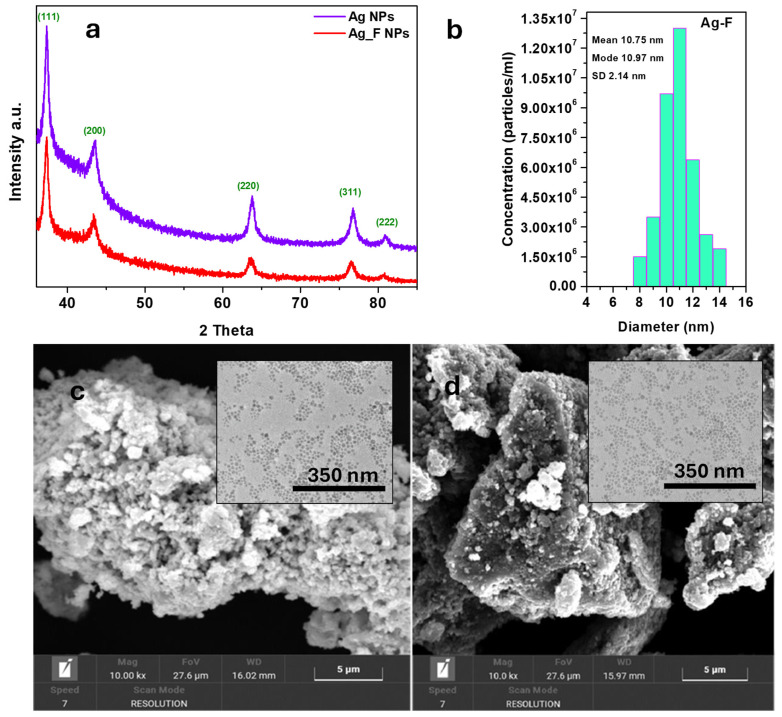
XRD spectra of Ag NPs and Ag-F NPs (**a**); NTA analysis of Ag-F NPs (**b**); SEM images of Ag NPs (**c**) and Ag-F NPs (**d**); TEM images of Ag NPs (inset in (**c**)) and Ag-F NPs (inset in (**d**)).

The XRD spectra of Ag NPs and Ag-F NPs are shown in Figure 1a. In both spectra, the characteristic diffraction peaks of silver appear at approximately 2θ = 37.5°, 43.4°, 63.8°, 76.7°, and 80.8°, corresponding to the Bragg reflections of the (111), (200), (220), (311), and (222) planes of Ag [40,41]. The results of the NTA analysis performed on the NPs are presented in Figure 1b. This analysis allows for the determination of the size distribution profile of small particles in suspension. As evidenced by the synthetic parameters reported in Figure 1b, the size distribution obtained through NTA indicates that the Ag-F nanoparticles have sizes below 15 nm.

The particle size determined through NTA analysis is in excellent agreement with the TEM data presented in this section. The reliability and precision of NTA in accurately assessing the size distribution of nanoparticles have been previously validated by several studies [42,43]. Compared to the commonly used dynamic light-scattering (DLS) technique, NTA provides direct visualization and tracking of individual particles, allowing for more accurate sizing and enhanced resolution of polydisperse samples [44].

The SEM images presented in Figure 1c,d provide valuable insight into the morphological characteristics of the analyzed samples, revealing the presence of quasi-spherical structures with dimensions reaching up to 1.0 µm ± 0.2. SEM analysis confirms that functionalization of the nanoparticles does not induce significant alterations in their overall morphology across different magnifications. SEM observations clearly revealed that Ag-F nanoparticles were uniformly dispersed throughout the leather-finishing surface. Such uniform distribution and shape consistency of silver nanoparticles have also been reported in similar composite systems, particularly when nanoparticles are integrated within polymeric matrices or natural fibrous materials, confirming the expected behavior and performance of our nanoparticles [16,20,45].

TEM images, shown in the insets of Figure 1c,d, correspond to the Ag NPs and Ag-F NPs samples, respectively. These images provide a more detailed visualization of the nanoparticle morphology, revealing their well-defined, nearly spherical shape and uniform size distribution, with an average diameter of approximately 11 nm ± 3.

**Figure 2:**
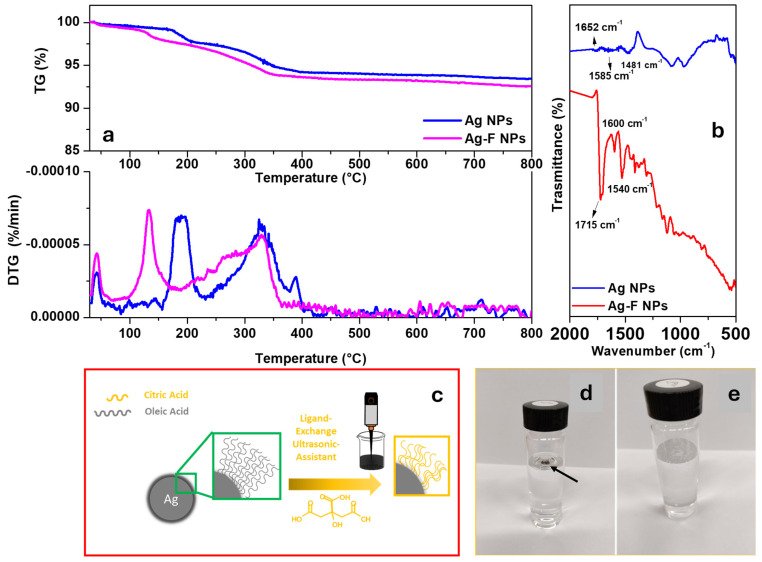
TG-DTG profiles of Ag and Ag-F NPs (**a**); FT-IR spectra of Ag and Ag-F NPs (**b**); schematic representation of the ligand-exchange process (**c**); and photographs of Ag NPs (**d**) and Ag-F NPs (**e**) dispersed in water.

To investigate the thermal stability of the synthesized materials, TGA was performed. Figure 2a presents the TG-DTG profiles of Ag NPs and functionalized Ag NPs under airflow conditions. In the blue thermogravimetric profile, corresponding to Ag NPs, two main weight loss events are observed, attributed to solvent evaporation at approximately 200 °C and the decomposition of organic chains (oleic acid) coating the nanoparticles at around 330 °C. The residual mass was 93 wt.%. In the magenta thermogravimetric profile, corresponding to functionalized Ag NPs, two weight loss events are evident, associated with water desorption at approximately 150 °C and the decomposition of citric acid between 230 and 340 °C. The residual mass was 92 wt.%.

TGA results highlighted distinct and reproducible thermal decomposition stages corresponding to solvent removal, decomposition of organic ligands (oleic acid), and further functionalization with citric acid. These findings align closely with previous reports on organically coated silver nanoparticles, which similarly showed well-defined thermal degradation steps at characteristic temperatures due to ligand desorption and organic decomposition [32].

FT-IR spectroscopy was conducted to analyze the nanoparticles in their as-synthesized state and after being coated with different organic ligands. Figure 2b displays the FT-IR spectra of Ag NPs coated with oleic acid after synthesis and those functionalized with citric acid (Ag-F NPs). As shown in the spectrum (blue profile), the silver nanoparticles in their post-synthesis state exhibit two vibrational bands at approximately 1585 cm^−1^ and 1652 cm^−1^, corresponding to the symmetric and asymmetric stretching modes of the -COO^−^ bond in oleic acid [33]. This confirms the conjugation of the carboxylate groups with the nanoparticle surface. The band at 1481 cm^−1^ is assigned to the symmetric stretching of the C-O bond in the COOH group of oleic acid [35]. Following functionalization with citric acid (red profile), the characteristic bands of citric acid at 1705 cm^−1^ and 1755 cm^−1^, attributed to the C=O stretching of the COOH group, disappear. Instead, two new bands appear at 1540 cm^−1^ and 1600 cm^−1^, indicating the complexation of citric acid on the silver nanoparticle surface, confirming successful surface modification [33]. The vibrational band at 1715 cm^−1^, characteristic of citric acid, remains visible, signifying the presence of a free carboxyl group [33], which enhances the hydrophilicity of the nanoparticles. The characteristic bands of oleic acid are no longer clearly detectable, likely due to complete replacement by citric acid molecules on the nanoparticle surface. The schematic representation of the ligand exchange illustrates (see Figure 2c) the replacement of oleic acid with citric acid on the surface of silver nanoparticles. This process enhances the nanoparticles’ hydrophilicity and stability, as the carboxyl groups of citric acid interact with the silver surface, replacing the oleic acid molecules [41]. The exchange is confirmed by FT-IR analysis, which shows the disappearance of oleic acid bands and the appearance of characteristic citric acid bands. Moreover, to confirm the successful ligand exchange, silver nanoparticles before and after modification with citric acid were dispersed in water. Figure 2d,e show the dispersion images of the non-functionalized silver nanoparticles and after functionalization with citric acid, demonstrating that the silver nanoparticles are well dispersed in an aqueous solution.

**Figure 3:**
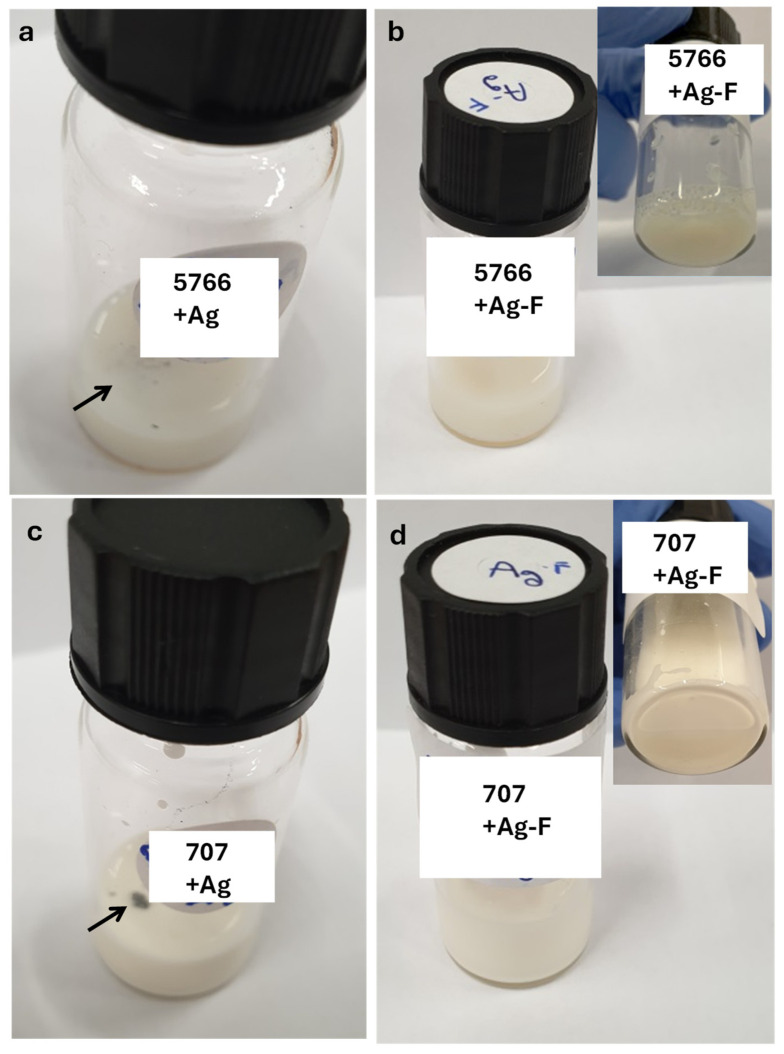
Dispersion of nanoparticles in 5766: (**a**) Ag NPs, (**b**) functionalized Ag NPs and dispersion of nanoparticles in 707, (**c**) Ag NPs, (**d**) functionalized Ag NPs.

A similar procedure was carried out to verify the compatibility of silver nanoparticles modified with citric acid with the PU5766 and 707 finishing inks. In this case as well, the nanoparticles exhibited excellent dispersion, with no sedimentation observed at the bottom (see Figure 3).

It should be noted that the polyurethane-based finishing agent PU5766 and the nitroemulsion-based finishing agent 707, although both functionalized with Ag-F nanoparticles, possess inherently different chemical compositions. Initially, both samples were collectively referred to as “Ag-F” for simplicity due to their comparable morphological and structural characteristics confirmed by SEM, TEM, and FT-IR analyses. However, these finishing agents present distinct chemical and functional characteristics, as clearly evidenced in subsequent performance analyses discussed in Section 3.1.2. The specific differences related to antibacterial efficiency, UV resistance, abrasion resistance, and micro-scratch resistance, arising from the distinct chemical nature of each finishing agent, will be explicitly analyzed and compared in the relevant subsequent section.

#### 3.1.2. Evaluation of Leather Properties

**Figure 4:**
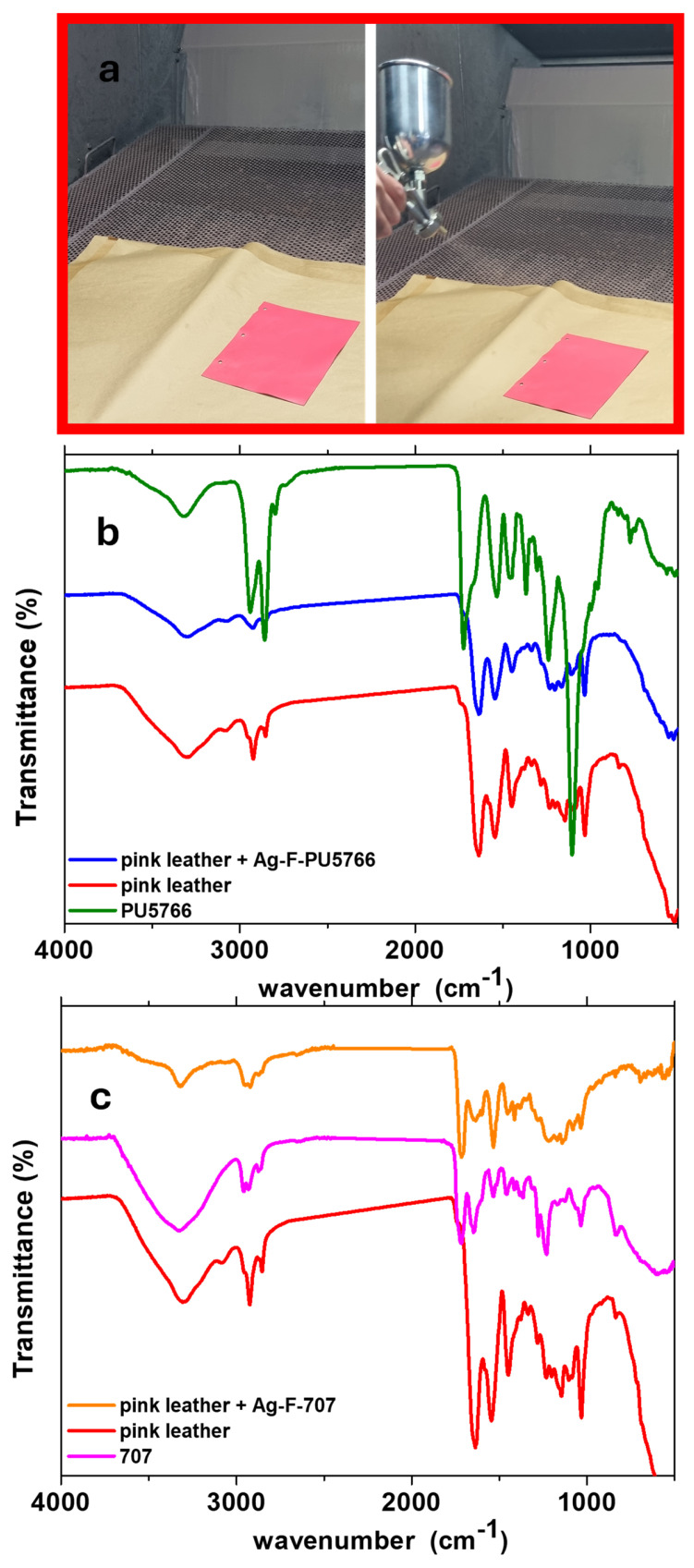
Photos of the leather-finishing process using a spray application (**a**); FT-IR spectra of untreated leather and finished leathers treated with Ag-F-PU5766 (**b**) and Ag-F-707; (**c**) comparison of these spectra with those of the PU5766 and 707 finishing inks.

Figure 4a presents images of the spray-coating process used to finish the leather samples with the prepared inks at DMD Solofra S.p.A. The FT-IR spectra of the pink leather samples, both with and without Ag-F NPs, are shown in Figure 4b and Figure 4c, respectively. The unfinished leather (pink) exhibits characteristic absorption bands associated with tanning materials. Specifically, a prominent band around 3302 cm^−1^ is attributed to the –O–H stretching vibration of phenolic compounds. Additionally, the bands observed between approximately 3000 and 2847 cm^−1^ correspond to the –CH_3_ stretching vibrations. Furthermore, three distinct bands at 1636, 1539, and 1443 cm^−1^ are assigned to the aromatic –C=C– stretching vibrations [34,46].

The FT-IR spectra of the leather after coating with Ag-F nanoparticles, using different finishing agents (PU5766 and 707), whose IR spectra are also provided for comparison, indicate that the presence of nanoparticles does not alter the composition of the leather substrate, either with or without the finishing blend. However, a certain attenuation of the bands can be observed in the FT-IR profile when the leather is coated with Ag-F NPs.

**Figure 5:**
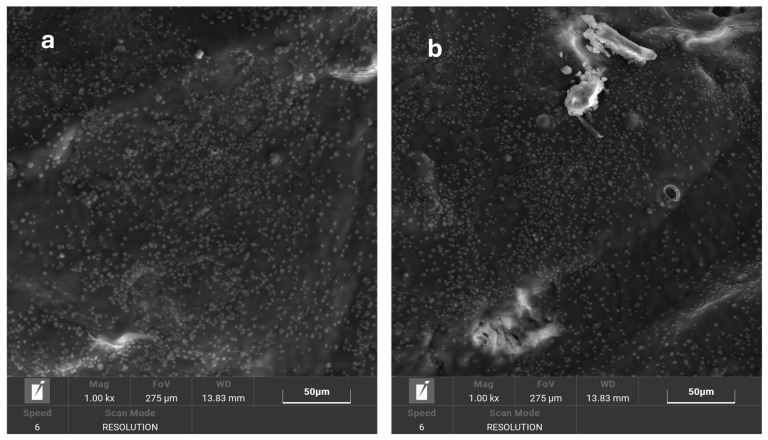
SEM image of pink leather finished with Ag-F-PU5766 finishing agent (**a**) and Ag-F-707 finishing agent (**b**).

SEM images were acquired to evaluate the appearance of the leather coating and the uniform dispersion of NPs. Figure 5a,b reveal a homogeneous distribution of silver nanoparticles on the surface of the leather samples finished with the two previously prepared inks. The NPs appear well dispersed, with no evident aggregation, indicating good compatibility between the functionalized silver nanoparticles and the finishing formulations. This uniform distribution suggests effective integration of the nanoparticles within the coating matrix, ensuring consistent functional properties in the finished leather.

The analysis of the results presented in Table 1 demonstrates enhanced antibacterial properties in the presence of silver nanoparticles (Ag NPs) within the finishing layer. This improvement can be attributed to the well-known antibacterial activity of silver nanoparticles. Their mechanism of bacterial inactivation involves the release of Ag^+^ ions, which penetrate the bacterial cell wall, inducing structural alterations in the cell membrane, such as increased permeability, ultimately leading to bacterial cell death [47,48,49,50]. Accelerated aging tests were conducted on the leather samples under investigation.

Specifically, Table 2 presents images of the leather samples after 24 h and 48 h of exposure to a xenon lamp at 50 °C. For comparison, a portion of the sprayed samples was covered during the test to prevent exposure. The results of the aging tests indicate that after 24 h and 48 h, the presence of silver nanoparticles (Ag NPs) influences the color stability of the leather. Notably, leather finished with Ag NPs exhibits improved resistance to fading. The enhanced UV protection provided by silver nanoparticles can be attributed to their incorporation into the finishing layer, which leads to high UV absorption and scattering. This effect is primarily due to the LSPR property of silver nanoparticles, which enables efficient interaction with UV radiation, thereby minimizing the degradation of the leather’s color and structure [16]. Color fading is a critical parameter for evaluating the durability and aesthetic stability of leather products, as it directly affects consumer perception and acceptance. This phenomenon is particularly relevant for leather used in automotive interiors, which are frequently exposed to intense solar radiation, leading to accelerated degradation. The incorporation of Ag NPs into finishing formulations represents a promising strategy for enhancing UV resistance and prolonging the lifespan of leather products. For this reason, accelerated aging tests were conducted on leather samples intended for the automotive sector.

Table 3 presents the test results, which indicate that the ∆E value (color change), along with all other color parameters, is lower for samples finished with silver nanoparticles (Ag NPs) compared to the untreated sample. This suggests that Ag NPs contribute to improved color stability.

Thanks to LSPR, silver nanoparticles enhance the brightness and vibrancy of colors without requiring additional dyes. Furthermore, key factors such as the content, size, size distribution, and shape of silver nanoparticles influence the LSPR band, allowing precise control over the coloration of fabrics [16]. The leather samples, both before and after finishing with silver nanoparticles (Ag NPs), were subjected to abrasion, wear, and micro-scratch tests to evaluate their mechanical resistance in comparison to a standard finished sample.

Table 4 presents the results of abrasion tests conducted using alcohol rubbing, the Martindale ball plate test, and the Taber test. In all cases, the samples incorporating Ag NPs exhibited a significant improvement in resistance compared to the control sample.

**Figure 6:**
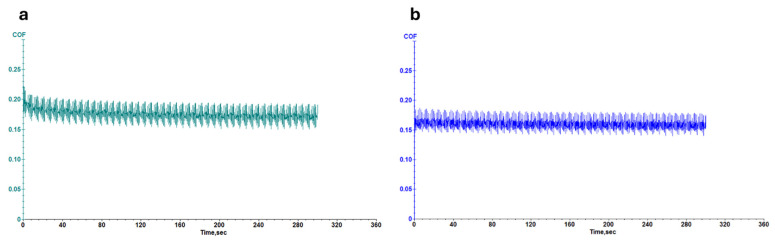
Wear measurement of pink leather with 707 finishing agent (**a**) and Ag-F-707 finishing agent (**b**).

**Figure 7:**
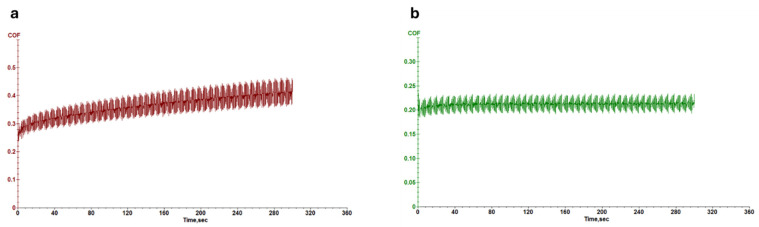
Wear measurement of leather with PU5766 finishing agent (**a**) and Ag-F-PU5766 finishing agent (**b**).

Figure 6 and Figure 7 illustrate the coefficient of friction (COF) as a function of time for leather samples coated with 707 finishing agents, both with and without Ag NPs (Figure 6), and PU5766 finishing agents, both with and without Ag NPs (Figure 7). A lower COF value during wear testing indicates enhanced resistance to surface friction. Notably, the samples containing Ag NPs exhibit a more stable COF over time, highlighting the exceptional durability of the finishing layer.

The progressive increase in COF is primarily associated with the surface degradation of the PU coating under mechanical stress, leading to an increase in surface roughness and, consequently, increased friction. Previous studies [52] have shown that unreinforced polymeric coatings tend to undergo significant wear, exposing surface asperities that enhance frictional interactions.

The instability in COF values can be related to the lack of homogeneity in the wear behavior of the PU coating. In the absence of reinforcing nanoparticles, the coating may exhibit a non-uniform distribution of stress, resulting in fluctuations in friction during the test. Previous research has demonstrated that non-reinforced polymeric coatings show heterogeneous wear patterns, which contribute to irregular tribological behavior [53].

The inclusion of functionalized silver nanoparticles (Ag-F NPs) in the coating, as shown in Figure 7b, significantly improves COF stability. The nanoparticles act as reinforcing agents, filling microdefects, reducing surface defects and enhancing the wear resistance of the coating, leading to more stable tribological performance.

Therefore, the increase and instability of COF observed in Figure 7a can be attributed to the lack of reinforcement in the PU coating. The addition of Ag-F NPs not only improves wear resistance but also minimizes surface irregularities, leading to a more stable frictional performance.

**Figure 8:**
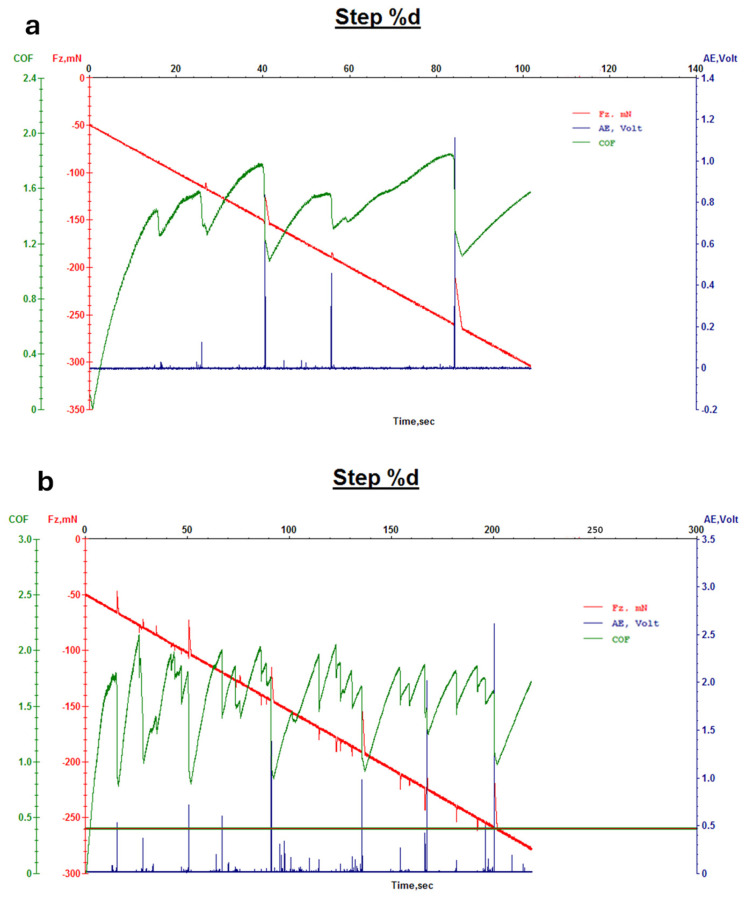
Micro-scratch measurement of leather with 707 finishing agent (**a**) and Ag-F-707 finishing agent (**b**).

**Figure 9:**
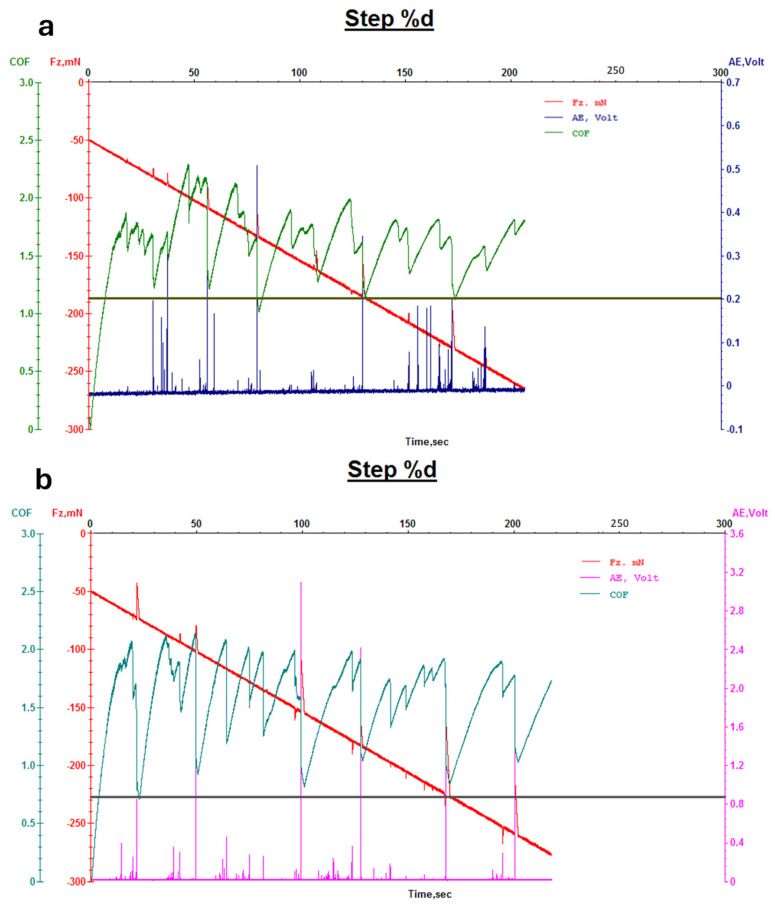
Micro-scratch measurement of leather with PU5766 finishing agent (**a**) and Ag-F-PU5766 finishing agent (**b**).

Furthermore, Figure 8 and Figure 9 depict the results of micro-scratch tests for leather samples finished with 707 finishing agents with and without Ag NPs (Figure 8) and PU5766 finishing agents with and without Ag NPs (Figure 9). In these tests, the addition of Ag NPs resulted in enhanced tangential forces (Fx), leading to an increased COF, which corresponds to improved micro-scratch resistance of the NP-based topcoat. The superior mechanical performance observed in Ag NP-functionalized topcoat layers can be attributed to the excellent dispersion of nanoparticles within the finishing ink.

This allows for the effective filling of micropores in the coating, thereby reducing surface defects. Additionally, when the coating surface is subjected to an external force, the high surface area of Ag NPs facilitates the adsorption of molecular chains, reducing local frictional stress and effectively enhancing wear resistance. Thus, the incorporation of Ag NPs not only improves the abrasion resistance of the finishing layer but also minimizes surface fracture areas, contributing to a more durable and mechanically robust leather coating [54].

## 4. Conclusions

This study demonstrated that the integration of functionalized silver nanoparticles (Ag-F NPs) into leather-finishing formulations results in a significant enhancement of antibacterial properties, UV resistance, and mechanical durability, making them highly suitable for demanding applications in automotive interiors, footwear, and leather goods.

Leather samples treated with Ag-F NPs exhibited a 90% reduction in microbial proliferation after 72 h. This effect is attributed to the controlled release of Ag^+^ ions, which actively disrupt bacterial cell membranes, preventing bacterial colonization and growth. The incorporation of Ag-F NPs thus provides an effective long-term antibacterial barrier, particularly beneficial for leather products frequently exposed to moisture and organic contaminants.

In addition to its antimicrobial properties, the Ag-F NP-based finishing layer significantly improved UV resistance and aging stability. After 48 h of accelerated aging under a Xenon lamp at 50 °C, treated leather samples exhibited 30% less color change (∆E) compared to control samples, demonstrating superior resistance to photodegradation. This property is particularly advantageous in automotive applications, where prolonged sun exposure can lead to fading and material degradation.

The introduction of Ag-F NPs had a profound impact on mechanical durability, as their uniform dispersion within the finishing layer not only reduces surface defects but also enhances wear resistance, ultimately improving the overall structural integrity of the coating.

These results pave the way for the industrial adoption of silver nanoparticles in leather-finishing treatments, offering advanced functionalities through a scalable and large-scale applicable process.

## Figures and Tables

**Table 1:** Results of antibacterial tests on leathers (TTC/MALT dipslide tests).

Sample	CFU/mL MALT			CFU/mL TTC		
	24 h	48 h	72 h	24 h	48 h	72 h
Leather with 707 finishing	//	<10^2^	10^3^	<10^2^	<10^2^	10^2^
Leather with 707+Ag finishing	//	//	<10^2^	//	<10^2^	<10^2^
Leather with 5766 finishing	//	10^3^	10^3^	<10^5^	10^5^	10^6^
Leather with 5766+Ag finishing	//	//	<10^2^	//	//	<10^2^

**Table 2:** Aging tests according to ISO 105 B02 standard.

Leather Sample	Exposure Time
Leather with 707 finishing	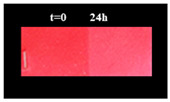
Leather with 707+Ag finishing	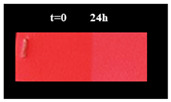
Leather with 5766 finishing	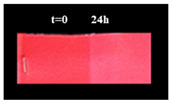
Leather with 5766+Ag finishing	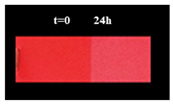

**Table 3:** Aging tests according to SAEJ 2412: evaluation of color fastness on leather samples intended for the automotive sector.

				Control Sample	Sample Ag-F NPs
Characteristics	Test Method	Requirements	U.M.	Results	Results
To artificial light 225 KJ/m^2^	SAEJ 2412 (acc. MS-JK 4000)	∆E ≤ 3.0 (for closed saloon car)	∆E	1.02	0.91
		Report ∆H, ∆C and L, a, b data	∆H ∆C ∆L ∆a ∆b	∆H: 0.24 ∆C: 0.13 ∆L: 0.98 ∆a: 0.04 ∆b: 0.27	∆H: 0.01 ∆C: 0.07 ∆L: 0.90 ∆a: 0.00 ∆b: 0.07
To artificial light 601 KJ/m^2^	SAEJ 2412 (acc. MS-JK 4000)	TBR	∆E	1.03	0.93
		Report ∆H, ∆C and L, a, b data	∆H ∆C ∆L ∆a ∆b	∆H: 0.48 ∆C: 0.42 ∆L: 0.81 ∆a: 0.09 ∆b: 0.63	∆H: 0.01 ∆C: 0.09 ∆L: 0.79 ∆a: 0.02 ∆b: 0.08

**Table 4:** Abrasive tests on leather finishing: control sample and with Ag-F NPs. Standards in the table column 2; U.M. cycles.

			Control Sample	Sample Ag-F NPs
Characteristics	Test Method	Requirements	Results	Results
To rub with alcohol	FCA 50444 [51]	TILL END OF LIFE ≥3 bleeding on cloth	Beginning break 10 CYCLES	Beginning break 70 CYCLES
Martindale ball plate	DIN EN ISO 17076-2	TILL END OF LIFE	Beginning break 1500 CYCLES	Beginning break 3000 CYCLES
TABER TEST_CS-10, 10 N	ISO 17076-1	TILL END OF LIFE Slight traces of abrasion are permitted. Not permissible: cracks in the finish such that the crust leather surface is visible	Beginning break 1000 CYCLES	Beginning break 2000 CYCLES

## Data Availability

The original contributions presented in this study are included in the article. Further inquiries can be directed to the corresponding author.
